# Diagnostic, Prognostic and Predictive Markers in Pediatric Germ Cell Tumors—Past, Present and Future

**DOI:** 10.3390/diagnostics12020278

**Published:** 2022-01-21

**Authors:** Michalina Jezierska, Ada Gawrychowska, Joanna Stefanowicz

**Affiliations:** 1 Department of Paediatrics, Haematology and Oncology, Faculty of Medicine, Medical University of Gdansk, 7 Debinki Street, 80-210 Gdansk, Poland; michalina.jezierska@gumed.edu.pl; 2 Department of Paediatrics, Haematology and Oncology, Clinical University Centre, 7 Debinki Street, 80-952 Gdansk, Poland; agawrychowska@uck.gda.pl; 3 Faculty of Health Sciences, Medical University of Gdansk, Maria Sklodowska-Curie Street 3a, 80-210 Gdansk, Poland

**Keywords:** germ cell tumors, markers, alfa-fetoprotein, beta-human chorionic gonadotropin, lactate dehydrogenase, miRNA

## Abstract

Germ cell tumors (GCTs) are a heterogenous group of neoplasms in children and young adults, in which serum tumor markers have been demonstrated to be highly sensitive diagnostic and monitoring tools. The known "old" serum biomarkers, alpha-fetoprotein (AFP), human choriogonadotropin (β-hCG) and lactate dehydrogenase (LDH), have some limitations in sensitivity and specificity. MIRNAs from the miR-371~373 (chromosomal locus 19q13.41) and miR-302/367 (4q25) clusters are universally over-expressed in malignant GCT tissue samples. The levels of miRNAs from these clusters are elevated in the serum. They seem to be highly sensitive and specific in malignant GCTs diagnosis and disease assessment during treatment and follow-up. The aim of our review was to present the role of serum tumor markers in the clinical staging, treatment monitoring and follow-up of pediatric patients with GCTs and show new possibilities. The serum levels of miRNAs seem to be a new, promising essential tool in the clinical management of GCTs.

## 1. Introduction

Germ cell tumors (GCTs) are infrequent in childhood, occurring at a rate of 2.4 cases per million children and representing approximately 3.5% of cancers diagnosed in children and adolescents younger than 15 years. However, GCTs are one of the most prevalent tumors in the adolescent and young adult age group, accounting for 13.9% of all the cancers among those aged 15 to 19 years [1]. The age distribution is bimodal, and small peaks occur—one from infancy to age four (type I GCTs), and the second after the onset of puberty (type II GCTs), with distinct clinical and molecular features in each age group [2].

GCTs derive from pluripotent precursor cells known as primordial germ cells and, hence, these tumors are characterized by phenotypic heterogeneity due to the largely retained embryonic pluripotency and aberrant somatic differentiation [2]. 

GCT precursor cells can differentiate to resemble extraembryonic structures, such as yolk sac (Yolk Sac Tumor (YST)) or placenta (choriocarcinoma (CC)). Type I tumors consist of teratomas, YSTs, or a combination of the two. Beginning at puberty, type II tumors arise and consist of seminoma, embryonal carcinomas (ECs), teratoma, YST, CC or combinations of different histologies. Type II testicular GCTs (TGCTs) are the most common cancer type in young adult and adolescent men. A precursor lesion known as germ cell neoplasia in situ (GCNIS) can be identified in type II tumors. In the 2016 World Health Organization classification of GCTs, type I and type II tumors are classified as “non-GCNIS associated” and “GCNIS associated”, respectively [2,3].

GCTs are a unique group of neoplasms due to their developmental potential determined by the latent potency state of their cells of origin, which are reprogrammed to omnipotent, totipotent or pluripotent stem cells. Oosterhuis and Looijenga proposed a new broad classification of GCTs, according to their developmental potential. The classification considers seven GCT types, each with distinct epidemiological and epigenomic features. Among them, two types are typical for children: type 0 and type I. Type 0 has the developmental potential for parasitic twins, including twins, occasionally, on aneuploidization, YST and somatic type malignancy (STM). It occurs prenatally or at birth in both sexes (F:M = 3:1), in sites where conjoined twins are attached: midline sacral region, retroperitoneum, abdomen, neck, face, orbit and other rare sites, with family history of monozygotic multiple pregnancies in 15% of cases. These GCTs are diploid, YST components near-diploid with a gain of 1q, 12p13 and 20q and/or a loss of 1p, 4 ad 6q. As for Type I, with the developmental potential of an immature teratoma, on aneuploidization, YST, rare cases of STM occur in neonates and children < 6 years of age in both sexes (F > M), but rarely beyond childhood, when located in the ovary, with a broad age range. Type I GCTs are located in the gonads and in the sacral region, retroperitoneum, abdomen, anterior mediastinum (thymus), neck, midline brain and other rare sites, with a family history of monozygotic multiple pregnancies in 15% of cases and karyotype, with a phenotype of a teratoma diploid and YST near the diploid, with a gain of 1q, 12p13 and 20q and/or a loss of 1p, 4 and 6q. Other types are typical for adolescents and adults [4,5]

GCTs in which the precursors remain undifferentiated, resembling primitive germ cells, are known as seminomas in males, dysgerminomas in females and germinomas when occurring in the central nervous system. GCTs are composed of numerous histologic subtypes that are independent of the presentation of clinical characteristics, tumor biology or clinical behavior, and vary with the site of origin, stage and age of the patient. Mature teratomas in infants are almost invariably diploid and benign, whereas those in the adult testis, with the same histologic features, are aneuploid and potentially malignant [2].

GCTs are classified into seminomatous germ cell tumors and non-seminomatous germ cell tumors (NSGCTs), the latter comprising the following malignant subtypes: yolk sac tumor, embryonal carcinoma and choriocarcinoma, in addition to teratomas, which show extensive somatic differentiation [2].

GCTs can present in both the gonadal and extragonadal sites. In general, extragonadal tumors comprise up to 40–55% of GCTs diagnosed in children aged younger than 4 years. In contrast, only 10–15% of GCTs diagnosed in adolescents are in extragonadal locations [6].

## 2. Serum Tumor Markers

### 2.1. α-Fetoprotein (AFP)

AFP, a product of the AFP gene located on chromosome 4q11-22, is a 70 k-Da glycoprotein. It is secreted by a yolk sac from the 10th week of pregnancy and then by the fetal liver and digestive system. The maximum concentration is achieved up to the third trimester of pregnancy and gradually decreases. During the first year of life, its level reduces by about four orders of magnitude and stabilizes at 5–10 ng/mL [7]. Table 1 cites the AFP reference values for children born in term according to the results of a study by Blohm et al. An interesting result of this study is the finding that the AFP half-life increased in the first year of life with postnatal age. Blohm et al. emphasize that for monitoring patients after the treatment of AFP-secreting tumors during the first year of life, a serial AFP assessment would be more appropriate than interpreting only the absolute values. This is a valuable clue in clinical work. AFP can be produced by malignant germ cell tumors (MGCTs) with the following histology: yolk sac tumors, embryonal carcinomas and immature teratomas. Al-Hussaini et al. also reported seven cases of elevated AFP in patients with Sertoli-Leydig cell tumors (SLCTs) that can be related to the foci of intestinal-type glands’ presence in some of these tumors [8]. AFP is currently used for an MGCT diagnosis, an assessment of the response to treatment and the stabilization of remission. AFP is a non-specific marker. Its increased level is seen in other types of neoplasms (e.g., hepatoblastoma, pancreato-blastoma, hemangioendothelioma) as well as in other diseases (e.g., chronic hepatitis, cirrhosis, cholestasis, ataxia telangiectasia and hereditary tyrosinemia) [7].

The following situations related to AFP seem to be important in oncological practice: elevated levels at the time of diagnosis, changes in levels during and after anti-neoplasm treatment and increasing AFP in specific clinical situations. There is no consensus in the literature regarding the prognostic role of elevated AFP at the time of diagnosis. The usefulness of AFP as a prognostic factor was demonstrated by Frasier et al. [9], who checked how using the IGCCC classification in pediatric malignant NSGCTs has an influence on prognostic factors. According to the IGCCC classification, the initial AFP level is one of the factors considered in dividing patients into the following three risk groups, respectively: for the group with good prognosis, <1000 ng/mL; for the group with an intermediate prognosis, ≥1000 and ≤than 10,000 ng/mL; and for group with a poor prognosis, >10,000 ng/mL. For the purpose of this study, 436 patients < 21 years old from intergroup studies POG 9048/CCG 8891 and POG 9049/CCG 8882 were used. In the final results, AFP > 10,000 ng/mL was defined as predictive of a poor outcome of event-free survival (EFS) and overall survival (OS). In turn, Baranzelli [10] reported the results of two consecutive TGM 85 and TGM 90 protocols for patients treated between January 1955 and December 1994 because of NSGCTs of the ovary. Secreting tumors (AFP ± β-hCG) were recognized in 49 patients in TGM 85, and 30 in TGM 90. According to this report, most of the failure occurred in patients with AFP > 15,000 ng/mL. In some research, a wider population including children and adults was analyzed. Furthermore, sometimes few tumor markers were analyzed together. Both situations may make it difficult to draw consistent conclusions for pediatric patients. For example, in studies of malignant ovarian GCTs performed by Murugaesu et al. [11] and Tangjitgmol et al. [12], Murugaesu et al., in 113 patients, aged 4–60 years, with stage IC to IV or stage IM, assessed the influence of age, histology, stage of disease and pre-treatment tumor markers on recurrence and OS. In this study, isolated AFP elevation turned out to be irrelevant, but both AFP and β-hCG being elevated was a predictor of OS. In the second study of 130 patients, aged 4 to 44 years, a preoperative increase in at least one of the tumor markers (AFP, β-hCG, LDH) was the only factor significantly affecting progression-free survival.

The most valuable analysis that negates the prognostic value of AFP at the time of diagnosis seems to be performed by Frazier et al. [13] in a summary of seven GCT trials conducted by the Children’s Oncology Group (United States) or the Children’s Cancer and Leukemia Group (United Kingdom) between 1985 and 2009. In a study of 519 patients, the researchers tried to review the risk factors and the risk classification based on these for extracranial GCTs in children. In multivariable analysis, AFP ≥ 10,000 ng/mL, which was found in 49% of the tested patients, was connected with a worse outcome, but this result was not statistically significant. This study identified a new group of risk factors: age ≥ 11 years, ovarian stage IV disease and extragonadal stage III to IV disease. Similar results concerning the prognostic usefulness of AFP were shown in a retrospective analysis of factors, such as age, stage, primary site, treatment and elevated alfa-fetoprotein, by Marina et al. [14]. The analysis included 165 children with extragonadal GCTs stages I–IV whom, between 1990 and 1996, were eligible for a trial of cisplatin dose intensity. Although AFP was elevated in 99 of them, it was irrelevant. Additionally, Calaminius et al. [15], who evaluated 71 children with malignant sacrococcygeal GCTs treated in the German Cooperative Protocols Maligne Keimzelltumoren (MAKEI) 83/86 and MAKEI 89, did not prove the prognostic role of elevated AFP at the moment of diagnosis.

AFP decline during anti-neoplasm treatment has a proven prognostic value in adults. The studies of this issue in the pediatric population are limited and the results are inconsistent. The first attempt to assess the prognostic value of AFP decline in pediatric patients was made by Fresneau et al. [16], who analyzed the results of the French TGM95 study. The study population included patients with NSGCTs aged < 18 years treated from January 1995 to December 2005. The patients were divided into the following three risk groups: low risk (n = 60, localized and complete resection, AFP < 15,000 ng/mL), intermediate (n = 65, incomplete resection, AFP < 15,000 ng/mL) treated with 3–5 cycles of VBP chemotherapy and high risk (n = 114, AFP > 15,000 ng/mL and/or disseminated disease) treated with 4–6 cycles of VIP chemotherapy. For an assessment of AFP decline in intermediate- and high-risk groups the following criteria were used: the predicted time to AFP normalization, the difference between AFP before and after the first cycle of chemotherapy and the difference between the observed and expected AFP decline area under the curve (based on AFP half-life). Contrary to adults, neither the predicted time to AFP normalization nor AFP changes were prognostic for these patients, whereas the study confirmed a negative prognostic of age ≥ 10 years, which is in line with analysis from the Malignant Germ Cell International Consortium (MaGIC) database.

A study that confirmed the prognostic value of AFP decline in pediatric patients was performed by O’Neill et al. [17]. In this study, data of patients treated on the Children’s Oncology Group (COG) protocol “A Phase III Study of Reduced Therapy in the Treatment of Children With Low and Intermediate Risk Extracranial Germ Cell Tumors” (COG AGCT0132) were analyzed. The study included patients diagnosed between 2003 and 2011. The age range was from 11 months to 21 years old (younger children were excluded because of potential difficulties in AFP level interpretation). The patients in the study were treated with PEB chemotherapy. The analysis took into account risk categories according to MaGIC (low risk, standard risk 1 and 2, poor risk). The Lange method was used to calculate the AFP decline. All 131 patients enrolled to the study had an elevated AFP. Most of the patients had a satisfactory marker decline (117 vs. 14 patients). The 3-year cumulative incidence of relapse in this group was 11% (38% for the group with unsatisfactory marker decline). This result was limited to 11 years old or older patients and patients with standard risk 2 disease (age ≥ 11 years and stage II–IV disease of the testis meeting International Germ Cell Cancer Consensus Group good-risk criteria, stage II–III disease of the ovary, or stage II disease of extragonadal sites).

Decline analysis of initially elevated AFP and β-hCG were performed in slightly older patients with extragonadal GCTs by Mazumdar et al. [18] and Ebi et al. [19]. In the first study, 189 patients, aged 15–60 years, were analyzed. The measurements of markers were performed between day 7 and 56, after two cycles of chemotherapy ended. The values between the first and seventh day were excluded because of the possibility of them increasing during the first days of therapy (an assumption resulting from the analysis of the literature). Among 181 patients, 152 had a satisfactory markers’ decline that was connected with the predicted improved complete response (CR) proportion and event free and overall survival. In the second study, 19 patients, aged 15–75 years, were examined. In all the patients, markers were re-measured 7 ± 2 days after starting chemotherapy. The two results were compared with each other. For patients with an increase in both markers, a decrease was defined as a decrease in both substances. A total of 11 patients had decreasing and eight patients increasing markers. Statistical analysis showed that patients with elevated marker values on the seventh day had a higher risk of progression and death. De la Motte Rouge et al. [20] also proved the prognostic significance of an early decline in AFP during chemotherapy, but only for ovarian yolk sac tumors. A study of 84 patients, aged 15–51 years, showed that an unfavorable early AFP decline was a significant negative predictive factor for OS. AFP measurements were made before and between the 18th and 21st day after the first cycle of chemotherapy.

AFP can be also used to monitor GCTs’ recurrence, but according to results found by Trigo et al. [21], it should not be used alone. It is interesting that it can also be useful in monitoring patients without pre-treatment AFP elevation at the first diagnosis. In a study conducted by Trigo et al., 798 patients, aged 15–78 years, with seminoma or NSGCTs in a gonadal or extragonadal localization were analyzed. A total of 125 had a recurrence recognized. Appropriately, 79 had an elevated AFP and β-hCG at the moment of the first diagnosis and 76 at the moment of recurrence. Of the patients, 68% had the same marker elevated in both clinical situations. The first symptoms of recurrence were elevated markers in NSGCTs and radiologic findings in seminomas. Keskin et al. [22], instead of AFP, tested the usefulness of tumor markers’ half-life (AFP, β-hCG) in relapsed NSGCTs. A total of 37 patients, aged 16–48 years, with relapse, and 28, aged 18–46 years, without relapse disease after first line treatment were enrolled into the study. AFP levels were measured before chemotherapy and after the second cycle. For purpose of the analysis, the International Germ Cell Cancer Collaborative Group risk classification system was used. There was no significance different between the groups in the AFP levels. The differences between groups occurred only in markers’ half-life analysis. In the case of AFP, the result was limited to groups with a good and intermediate prognosis.

The metastatic GCTs are a group undoubtedly requiring separate AFP role analysis. Seidel et al. [23] analyzed 707 such patients with an intermediate prognosis, aged ≥ 16 years. They confirmed that the baseline AFP with a cut-off value of 6200 IU/L is an independent prognostic factor for OS and is significantly associated with progression-free survival, which may be helpful to stratify these patients. Kobayashi et al. [24] and Spiess et al. [25] conducted studies on AFP and metastatic GCTs, but in the surgery interventions context. Kobayashi et al. attempted to assess the significance of elevated AFP levels before the resection of residual tumors in patients with extragonadal GCTs and metastatic NSGCTs after at least one line of treatment. Based on an analysis of 68 patients, aged 3–59 years, they found that the number of viable malignant cells in the residual mass in patients with a normal (5–10 ng/mL) and mildly elevated AFP (10–30 ng/mL) was similar, while all the patients with AFP > 30 ng/mL had viable malignant cells. This study proved that the mild elevation of AFP may be caused by residual malignances as well as by benign causes (e.g., post chemotherapy liver dysfunction) and is not always connected with poor prognosis. In such cases, the evaluation should be careful and individual for each patient. Spiess et al. performed a study of 236 patients, aged 15–58 years, with metastatic NSGCTs who needed a retroperitoneal lymph nodes dissection. Before a surgery procedure, the patients had an AFP measurement and then an assessment of the AFP predictive value for disease-specific survival, and it was >9 ng/mL.

Patients with sex development and additional Y chromosome material are in a specific situation because they have a higher risk of GCTs, especially gonado-blastoma and dysgerminoma. Huang et al. [26] described 292 patients with disorders of sex development, aged 15–75 years, who had undergone a bilateral gonadectomy. An assessment of tumor markers was performed here in cases with a pelvic mass found before surgery. An elevated AFP was seen in two patients with yolk sac tumors diagnosis and normalized after surgery. Therefore, AFP can be helpful for monitoring patients with a higher risk of GCTs’ development.

### 2.2. β-chorio-gonadotropin (β-hCG)

β-hCG, a glycoprotein composed of α- and β-peptide subunits, is normally synthesized during pregnancy by the syncytio-trophoblasts of the placenta to maintain the viability of the corpus luteum. The α-subunit is similar to α-peptides of other hormones, such as the luteinizing hormone, follicle-stimulating hormone and thyroid-stimulating hormone. The β-subunit is antigenically distinct, serving as the basis for the method of serum assays [27]. Minute amounts of β-hCG, less than 5 IU/mL, are detected in the serum of healthy adults; the serum half-life time of β-hCG is 24–36 h. The elevation of serum β-hCG in patients with GCTs implies the presence of clones of syncytio-trophoblasts, such as choriocarcinoma, or of syncytio-trophoblastic giant cells, found frequently in germinomas (pure seminomas or dysgerminomas) and occasionally in adult embryonal carcinoma [28]. Syncytio-trophoblastic cells can be found in any GCT tumor’s component, particularly in the testis. Rising or persistently elevated levels of β-hCG usually indicate residual or progressive disease, but as with AFP, other explanations may exist and warrant investigation [11].

Infantile choriocarcinoma is a highly malignant germ cell sub-entity thought to originate from the placenta. Children suffering from this tumor become symptomatic at a median age of 1 month. Elevated β-hCG is typical. The natural disease course is rapidly fatal [29].

There are possible iatrogenic hypogonadisms secondary to bilateral orchiectomy, oophorectomy or chemotherapy associated with rising levels of serum β-hCG because of an increase in the luteinizing hormone that results in immunologic cross-reactivity [2].

#### β-chorio-gonadotropin (β-hCG) and Male GCTs

In prepubertal GCTs, teratomas comprise a majority of GCTs, accounting for up to 40% of testicular tumors. When a malignant histology is present, pure yolk sac tumors are most common. In contrast, in adolescents and young adults, most GCTs are mixed non-seminomas, with tumors consisting of more than one malignant histology and a higher proportion of embryonal carcinoma and choriocarcinoma. The following serum tumor markers, AFP, β-hCG and LDH, play important diagnostic roles, and higher levels correlate with an increased burden of disease and are used for risk assignment as well as for treatment assessment [18].

It is very important to underline that the IGCCC separated men into good, intermediate and poor risk strata on the basis of histology (seminoma/non-seminoma), tumor markers levels, site (testicular/other) and the presence of non-pulmonary visceral metastases. This classification takes into account the tumor markers’ level and patients with β-hCG ≥ 5000 IU/L and ≤50,000 IU/L have an intermediate prognosis, whereas those with β-hCG > 50,000 IU/L have a poor prognosis. In this group, 5-year survival was only 48% [30].

### 2.3. Lactate Dehydrogenase (LDH)

Lactate dehydrogenase is one of the serum markers that is often elevated in patients with GCTs. However, LDH is a very non-specific marker and has limited clinical value. LDH is a glycolytic cellular enzyme released from the cells of every tissue of the body at apoptosis. It can be elevated in all kinds of GCTs and other malignancies as well as in non-malignant conditions such as chronic liver disease, stroke or hemolytic anemia. 

LDH level is one of the prognostic factors used to categorize adult men with non-seminomatous metastatic testicular GCT into three prognostic groups (good, intermediate and poor prognosis) according to the IGCCC criteria. An LDH level below 1.5× its upper limit of normal range (ULN) prior to the initiation of chemotherapy is associated with good prognosis, an LDH level between 1.5× and 10× the ULN with intermediate prognosis and an LDH level above 10x the ULN with poor prognosis. Frazer et al. [9] conducted a study to evaluate if the IGCCC criteria are also prognostic for pediatric patients with GCTs. One of the criteria applied was serum tumor markers levels at diagnosis. LDH was ≤1.5× UNL in 191 patients and >1.5× UNL in 148 patients. Only 10 children had LDH > 10× UNL. An LDH level ≥ 1.5 ULN was identified to be one of the prognostic variables for overall survival in these patients. However, a multivariate analysis revealed that it was not independently prognostic of a poor outcome.

LDH levels may be helpful in detecting seminomatous germ cell tumors in patients with disorders of sex development. Huang et al. [26] investigated 292 phenotypic female patients ranging in age from 8 to 42 years with Y-chromosome sequencies in their genomes. All the patients underwent a bilateral gonadectomy. Serum tumor markers including LDH were preoperatively assessed in the patients with a pelvic mass detected by physical examination or ultrasound. A total of 45 patients (26 of whom were below 20 years of age) were confirmed to have gonadal germ cell tumor by histopathological examination. An elevated LDH level ranging from 253 to 399 U/l (with a reference range of 0 to 250 U/l) was found in three of six dysgerminoma patients and three of five seminoma patients. However, in all of the six cases, the diameters of the tumors were above 5 cm and minor dysgerminoma/seminoma are inclined to present a normal LDH level. Capito et al. [31] described six patients aged 7 to 17 years with 46XY pure gonadal dysgenesis, who developed ovarian dysgerminoma. LDH was preoperatively measured in three of these and occurred to be elevated to a rate of 1917 IU/L, 2739 IU/L and 5570 IU/L (with a normal range under 350 IU/L). However, all of these tumors measured above 15 cm, which could lead to an LDH elevation because of a higher tumor burden. LDH is a marker of increased cellular turnover and is not specific for these tumors. 

Serum tumor markers can be also useful in differentiating benign from malignant ovarian tumors in children. Stankovic et al. [32] investigated 53 girls with ovarian tumors managed operatively. The patients’ age at the time of surgery ranged from 9 months to 19 years. Serum tumor makers (AFP, β-hCG, LDH, CA 125, estradiol or testosterone) were elevated in 17 cases, including 13 of the 18 patients with malignant tumors and four of the 35 patients with benign tumors. LDH was found to be elevated in two of the four dysgerminoma patients, in one patient with a yolk sac tumor and in one patient with a juvenile granulosa cell tumor.

Table 2 presents the most important studies of the traditional markers in GCTs (Table 2).

### 2.4. Serum microRNAs as New Biomarkers in GCTs

The gene expression in GCT is regulated, in part, by DNA and histone modifications, and the epigenetic profile of these tumors is characterized by genome-wide demethylation, except non-seminomas. In addition, it was recently discovered that the mechanism of post-genomic gene expression regulation involves small non-coding RNAs, predominantly micro-RNA (miRNA). 

MiRNAs are short, non-protein coding RNAs that are highly stable and suitable for diagnosis and disease-monitoring. MiRNAs from the miR-371~373 (chromosomal locus 19q13.41) and miR-302/367 (4q25) clusters are universally over-expressed in malignant GCT tissue samples. The levels of miRNAs from these clusters are elevated in the serum. They seem to be highly sensitive and specific in malignant GCTs diagnosis and disease assessment during treatment and follow-up. 

Targeted miRNA-based blood tests for miR-371-3 and miR-367 clusters are currently under development and hold great promise for the future.

In some patients, miRNA-based tests may be even more sensitive than the classical serum tumor markers—β-hCG, AFP and LDH—which are currently used in the clinic [36]. Dieckmann et al., in a prospective, multicentric study with the participation of 616 patients with testicular GCTs, confirmed that the M371 test outperforms the classic markers of GCT with both a sensitivity and a specificity greater than 90%. AFP, β-hCG and LDH had sensitivities of less than 50% in seminoma and slightly higher sensitivities in non-seminoma [37].

Plaza et al. [38] assessed miR-371a-3p, miR-373-3p and miR-367-3p as serum biomarkers in metastatic testicular GCTs and confirmed that high miRNA levels at diagnosis are associated with worse clinical outcomes and can assist in the early diagnosis of relapses.

Interestingly, the characteristic miRNA profiles are similar in the GCTs that arise in children and adults, and are detectable in all the histological subtypes and components, except teratoma [39]. Murray et al. confirmed that pediatric malignant GCTs show biological differences from their adult counterparts at a genomic and protein-coding transcriptome level, whereas they both display very similar microRNA expression profiles [39].

Other miRNA expression changes reflective of different malignant-GCT subtypes also occur in the tissues, such as the overexpression of microRNAs from the “chromosome-19-microRNA-cluster” (C19MC or miR-515~526) in embryonal carcinoma. C19MC is co-located within 100 kb of the miR-371~373 cluster and occurs uniquely in primates. 

Murray et al. identified a serum panel of choriocarcinoma-specific “chromosome-19-microRNA-cluster” (C19MC) microRNAs that were highly elevated at diagnosis but fell rapidly upon treatment and normalized before the second full chemotherapy course. They also re-confirmed the serum elevation of the previously identified malignant-GCT marker miR-371a-3p at diagnosis. The authors concluded that circulating microRNA markers reflected choriocarcinoma disease activity more accurately than serum β-hCG and real-time knowledge would have assisted clinical decision making [40].

Almstrup et al. assessed the classic serum tumors markers (AFP, β-hCG and LDH) and compared them with miRNA-based markers in diagnosing and in the follow-up monitoring and prediction of the relapse of GCTs (Table 3) [41]. Table 3 presents the comparison of classic serum tumour markers with miRNA-based markers [41]. MiR-371a-3p is the most consistent marker and exhibits >90% diagnostic sensitivity and specificity in TGCT. At first presentation, AFP, β-hCG and LDH levels are elevated in only 26–34%, 38–47% and 33–44%, respectively, of all patients with TGCTs. The authors emphasized the current limitations and challenges of the miRNA-based biomarkers. miRNA biomarkers do not detect mature teratoma, and they are not useful in diagnosing and follow-up monitoring germ cell neoplasia in situ (GCNIS) [41,42,43,44]. The last systemic review by Leão et al. confirms the potential of circulating miRNA levels, particularly of miR-371a-3p, for incorporation in clinical practice [42]. Lobo et al. confirmed the feasibility of the hypermethylated *RASSF1A_M_* detected in circulating cell-free DNA as a non-invasive diagnostic marker of testicular germ cell tumors, combined with miR-371a-3p. Lobo et al. described a novel droplet digital polymerase chain reaction method for quantitatively assessing *RASSF1A_M_* in liquid biopsies. Both miR-371a-3p (sensitivity = 85.7%) and *RASSF1A_M_* (sensitivity = 86.7%) outperformed the combination of AFP and β-hCG (sensitivity = 65.5%) for TGCT diagnosis. *RASSF1A_M_* detected 88% of teratomas. The combination of both biomarkers detects disease (including teratoma) with a sensitivity of 100% [45]. 

### 2.5. Tumor Markers as a Method of Relapse Detection in Children, Adolescent and Young Adults and Adults

Fonseca et al., based on the COG AGCT0132 trial, confirmed that tumor marker elevation is a highly sensitive method of relapse surveillance, at least among children and adolescents with tumor marker elevation at their initial diagnosis. Fonseca et al. analyzed a group of 284 patients enrolled in the American trial with low and intermediate risk GCTs. Only seven patients had normal tumor markers at their initial diagnosis, and none of them experienced a relapse. At a median follow-up of 5.3 years, 48 (16.9%) patients had experienced a relapse; of these, 47 of the 48 relapses were detected by tumor markers’ evaluation. Among these patients, 31 had AFP elevation, two had β-hCG elevation and 15 had both AFP and β-hCG elevation at diagnosis. At relapse, 39 patients (81%) had AFP elevation, one (2%) had β-hCG elevation, seven (15%) had both tumor markers elevated and one did not have available tumor marker data. The COG researchers concluded that eliminating exposure to imaging with ionizing radiation may enhance the safety of relapse surveillance in patients treated for MGCTs [46]

In contrast are the conclusions of the last study carried out by Chakiryan et al. The purpose of this study was to conduct a systematic review of the currently available evidence evaluating the reliability of serum tumor markers as a test for the diagnosis of recurrence in patients with clinical stage I non-seminomatous embryonic tumors under active supervision. This systematic review was conducted in accordance with PRISMA guidelines, with no language or data restrictions and includes studies that readily identified the tumor marker status of patients with clinical stage I non-seminomatous germ cell tumors who had a recurrence of active surveillance, with marker positivity as the primary outcome at the time of recurrence. The researchers identified 2157 studies, and ultimately included 37 studies with 8545 patients with clinical stage I NSGCTs managed by active surveillance, and 2254 finally relapsed. Serum tumors’ markers were elevated in 28–75% of the patients at the time of recurrence and were the only indication of recurrence in 4–39%. Chakiryan et al. concluded that in patients with clinical stage I NSGCTs managed by active surveillance, the use of serum tumor markers cannot obviate the need for CT, and to limit radiation exposure more reliable markers are needed [47].

## 3. Summary

Serum tumor markers, the “onco”-fetoproteins (AFP and β-hCG), are helpful in the diagnosis and monitoring of GCTs, as well as in the detection of residual or progressive disease. They are included in commonly used staging manuals. AFP and β-hCG are secreted by non-seminomatous tumors, yolk sac tumors and syncytio-trophoblasts of choriocarcinoma, whereas LDH is also secreted by seminomas.

In adult men with testicular cancer, elevations of these markers were shown to portend (announce) a poor prognosis [30]; a rate of decline has been demonstrated to be a sensitive marker of response to therapy [18].

However, the following conventional biomarkers, AFP, β-hCG and LDH, have limited utility for the diagnosis and follow-up of malignant GCTs. LDH is very non-specific. The detection and secretion of AFP and β-hCG in body fluids is restricted to specific subtypes, with levels raised predominantly in tumors containing a yolk sac tumor and choriocarcinoma histopathology, respectively [48]. 

Repeatedly, the interpretation of elevated AFP and β-hCG levels creates many difficulties in clinical practice. The problem of interpretation in pediatric GCTs includes clinical data (age of the patients—children), the long half-lives of AFP (~5–7 days) and β-hCG (~12–36 h) and their limited specificity and histopathology [48].

Embryonic miRNAs show great potential for the clinical management of testicular GCTs. The use of these miRNAs could potentially identify patients with a worse prognosis [34,35,36,37].

This review has some limitations. The number of studies of GCT markers in the pediatric population is very small. Therefore, it is impossible to conduct statistical analyses of the studies and data found. The search difficulties are probably related to the different keywords used by different authors.

## 4. Materials and Methods

### 4.1. Search Strategy

The search was conducted following the Preferred Reporting Items for Systematic Review and Meta-Analysis (PRISMA) guidelines (Moher D). 

Studies investigating the serum markers in malignant extracranial GCTs in children and young adults were identified via a literature search of PubMed. Studies with a research population of patients aged 0–18 were considered as “pediatric”.

Our overall search strategy included the following basic heading: germ cell tumors and markers; and the following subheadings: alfa-fetoprotein, beta-human chorionic gonadotropin, lactate dehydrogenase and miRNA. We searched each subheading with basic headings independently and separately. 

Filters applied were English, Child: birth—18 years, time period—from 1 January 2000 to 31 October 2021.

The resulting titles and abstracts were screened for relevance and inclusion and exclusion criteria, and the resulting articles were reviewed.

### 4.2. Study Selection

The inclusion criteria for this review were articles focusing on the markers in GCTs in the pediatric population. However, when the study included very important and new data on markers, we decided not to exclude articles from our review that included an adult or young adult population. The exclusion criteria were letters to the editors, case reports, non-English literature, non-human studies and studies of other cancers.

## 5. Conclusions

In conclusion, the results of the current review describe the role of serum tumor markers in GCTs’ diagnosis, risk stratification, monitoring and prognosis in the past, present and future and shows the need to search for new markers to achieve the best risk stratification. Future international collaboration is necessary for the best treatment possibilities, regardless of where the patient is diagnosed and treated.

## Figures and Tables

**Table 1:** Serum AFP value of term babies according to Blohm et al., (1998), *Pediatr. Hematol. Oncol*.

Age (Days)	AFP Mean (ng/mL)	AFP 95.5% Interval (ng/mL)	Half-Life (Days)
0	41,687	9120–190,546	
1	36,391	7943–165,959	
2	31,769	6950–144,544	
3	27,733	6026–125,893	
4	24,210	5297–109,648	
5	21,135	4624–96,605	5.1
6	18,450	4037–84,334	
7	16,107	3524–73,621	
8–14	9333	1480–58,887	
15–21	3631	575–22,910	
22–28	1396	316–6310	
29–45	417	30–5754	14
46–60	178	16–1995	
61–90	80	6–1045	28
91–120	36	3–417	
121–150	20	2–216	42
151–180	13	1.25–129	
181–720	8	0.8–87	no correlation

AFP—alpha-fetoprotein.

**Table 2:** Summary of the most important studies on AFP, β-hCG and LDH in GCT used in this review.

Marker	Literature	Study Population	Results and Conclusions
Initial AFP Elevation	Blohm et al., (1998), *Pediatr. Hematol.* *Oncol.*	Infants up to the age of 2 years (term and premature babies born), excluding children with factors connected with elevation of AFP	AFP values higher than in previous reports AFP values higher in term babies than in premature born AFP half-life in infants increased with postnatal age In infants after AFP-secreting tumors treatment, serial AFP assessment is advisable
	Frazier A.L. et al., (2008), *Pediatr. Blood Cancer*	Patients ≤ 21 years with MGCTs from intergroup studies POG 9048/CCG 8891 and POG 9049/CCG 8882	Initial AFP > 10,000 ng/mL is an adverse prognostic factor
	Baranzelli et al., (2000), *Eur. J. Cancer*	Patients < 18 years with NSGCTs of the ovary from TGM 85 and TGM 90	Initial AFP > 15,000 ng/mL is an adverse prognostic factor
	Murugaesu N. et al., (2006), *J. Clin. Oncol.*	Patients from 4 to 60 years with MGCTs of the ovary	Initial elevated AFP and β-hCG are adverse prognostic factors
	Tangjitgmol S. et al., (2010), *Acta Obstet. Gynecol.* *Scand.*	Patients from 4 to 44 years with MGCTs of the ovary	Preoperative elevated at least one of the tumor markers (AFP, β-hCG, LDH) is an adverse prognostic factor
	Frazier A.L. et al., (2015), *J. Clin. Oncol.*	Patients from 7 MGCTs trials conducted by the Children’s Oncology Group or the Children’s Cancer and Leukemia Group	Initial AFP > 10,000 ng/mL is not a prognostic factor New prognostic factors (age ≥ 11 years, ovarian stage IV disease and extragonadal stage III to IV disease)
	Marina et al., (2006), *J. Clin.* *Oncol.*	Patients < 18.5 years with MGCTs from trial of cisplatin dose intensity	Initial elevated AFP is not a prognostic factor Age ≥ 12 years and thoracic site are adverse prognostic factors
	Calaminus G. et al., (2003), *J. Clin.* *Oncol.*	Children with sacrococcygeal MGCTs treated in the German Cooperative Protocols Maligne Keimzelltumoren (MAKEI) 83/86 and MAKEI 89	Initial elevated AFP is not a prognostic factor
AFP Decline	O’Neill A.F. et al., (2019) *Cancer*	Patients from 11 months to 21 years treated on Children’s Oncology Group (COG) protocol COG AGCT0132	AFP decline is associated with cumulative incidence of relapse in pediatric patients treated for MGCTs
	Mazumdar et al., (2001), *J. Clin.* *Oncol.*	Patients from 15 to 60 years with elevated AFP/β-hCG, treated with platinum-based chemotherapy	Rate of AFP/β-hCG decline during chemotherapy is a prognostic factor
	Ebi H. et al., (2003), *Cancer*	Patients from 15 to 75 years with MGCTs treated at the National Cancer Center Hospital East, Japan	Transient AFP/β-hCG elevations on day 7 is an adverse prognostic factor
	De la Motte Rouge T. et al., (2016), *Gynecol. Oncol.*	Patients from 15 to 51 years with ovarian YSTs cases at Gustave Roussy (Villejuif, France)	Early AFP decline is a good prognostic factor The ability to calculate AFP decline using free calculator (available on the Gustave Roussy website)
	Fresneau et al., (2018), *Eur. J. Cancer*	Patients ≤ 18 years with NSGCTs from TGM 85 and TGM 90	The predicted time to AFP normalization is not a prognostic factor Age ≥ 10 years is an adverse prognostic factor
β-hCG	International Germ Cell Cancer Collaborative Group (1997), *J. Clin. Oncol*	5202 patients with non-seminomatous GCT (NSGCT) and 660 patients with seminoma from 10 countries, <20 years—562 (13%)	AFP > 10,000 ng/mL, β-hCG > 50,000 IU/L (10,000 ng/mL) and LDH > 10x upper limit of normal are factors of poor-prognosis in non-seminoma
	Schneider, D.T. et al., (2001), *Pediatr. Hematol. Oncol.*	Review and summary of the experience of the German cooperative protocols for non-testicular germ cell tumors (MAKEI) on the use of AFP and β-hCG for diagnostic evaluation in pediatric oncology	Benign disorders such as CKD, systemic lupus erythematosus-Choriocarcinoma -Pure germinomas—testicular seminoma, ovarian dysgerminomas -Hepatoblastoma, hepatocarcinoma
	Blohm, M.E.G. et al., (2004), *Eur. J. Pediatr.*	30 children with infantile choriocarcinoma	Serum β-hCG levels were universally elevated in all 19/19 tested infants
	Frazier A.L. et al., (2008), *Pediatr. Blood Cancer*	Patients ≤ 21 years with MGCTs from intergroup studies POG 9048/CCG 8891 and POG 9049/CCG 8882	β-hCG level was not found to be prognostic of outcome, the adult cut point is not informative for pediatric patients
	Terenziani M. et al., (2017), *Pediatr.* *Blood Cancer*. [33]	The Associazione Italiana Ematologia Oncologia Pediatrica (AIEOP) experiences with malignant ovarian GCT, 03.2004–12.2015, 77 patients < 18 years old (median age 11.8 years (range 1.8–17.2)	β-hCG at diagnosis [IU/L] ≤5000—13 5000–50,000—6 ≥50,000—3
	Terenziani M. et al., (2018), *Urol. Oncol.* [34]	The Associazione Italiana Ematologia Oncologia Pediatrica (AIEOP) experiences with malignant testicular GCTs in children and adolescents, 03.2004–12.2015, 99 patients ≤ 18 years old (median age 15.4 years (range 0.5–17.8)	β-hCG at diagnosis [IU/L] ≤5000—44 5000–50,000—6 ≥50,000—0
	Fonseca, A. et al., (2019), *J. Clin. Oncol.*	Children’s Oncology Group, low-risk and intermediate-risk MGCTs patients (284 patients)	Relapses detected by: AFP elevated—39 β-hCG elevated—1 AFP and β-hCG elevated—7
	D’Angelo P et al., (2021), *Pediatr. Blood Cancer* [35]	The Associazione Italiana Ematologia Oncologia Pediatrica (AIEOP) experiences with malignant sacrococcygeal GCTs in children and adolescents, 01.2004–05.2017, 45 patients, 35 were females, age at diagnosis—median 1.6 years (range 1 day to 3.6 years); stage IV-26	β-hCG Normal—38 Not done—2 High—5, only neonates (may be due to the physiological β-hCG maternal circulation)
LDH	Frazier A.L. et al., (2008), *Pediatr. Blood Cancer*	Patients ≤ 21 years with MGCTs from intergroup studies POG 9048/CCG 8891 and POG 9049/CCG 8882	LDH level ≥ 1.5 ULN is not independently prognostic of poor outcome
	Huang, H. et al. (2017), *Clin. Endocrinol. (Oxf).*	292 phenotypic female patients ranging in age from 8 to 42 years with disorders of sex development containing Y chromosome or Y-derived sequence undergoing bilateral gonadectomy	LDH was elevated in 3/6 dysgerminoma patients and in 3/5 seminoma patients. All of these 6 tumors were >5 cm in diameter; therefore, LDH elevation could be caused by big tumor mass and LDH is not considered to be specific for GCTs
	Capito, C. et al., (2011), *J. Pediatr. Urol..*	6 patients aged 7 to 17 years with dysgerminoma associated with 46, XY pure gonadal dysgenesis	LDH strongly elevated in 3/6 patents (in 3 patients LDH level was not tested). Although, LDH elevation could be caused by big tumor mass (tumors > 15 cm in diameter)
	Stankovic, Z.B.et al., (2006), *J. Pediatr. Endocrinol. Metab*	53 female patients ranged 13 months to 19 years surgically treated for 59 ovarian tumors, including 6 bilateral	Serum tumor markers (AFP, β-hCG, LDH) can be useful in preoperative differentiating benign from malignant ovarian tumors

AFP—alpha-fetoprotein, β-hCG—beta-human chorionic gonadotropin, LDH—lactate dehydrogenase, MGCTs—malignant germ cell tumors, NSGCTs—non-seminomatous germ cell tumors, YSTs—yolk sac tumors.

**Table 3:** Classic serum GCTs markers, miRNA-based markers and histological subtypes [Almstrup 2020].

	Detection Rates in Testicular GCTs							Extragonadal	Detection Rates in Non-Testicular GCTs
	Germinoma (DysgerMinoma/ Seminoma)	Non- Seminoma	YST	Embryonal Carcinoma	Non- Gestational Choriocarcinoma	Teratoma	Mixed Tumors		
AFP	<3%	60–70%	>95%	40%	<5%	20–25%	variable	variable	12%
β-hCG	18–31%	53%	<5%	25%	>95%	~10%	variable	variable	14%
LDH	29%	39%	10%	20%	20%	<5%	variable	variable	high
miR-371a-3p	87%	94%	>90%	>90% ^a^	>90%	<5% ^a^	~90%	>90% ^a^	6%
miR-373-3p	70%	80%	+	+	+	<5% ^a^	~60% ^a^	+	11%
miR-367-3p	79%	85%	+	+	+	<5% ^a^	75% ^a^	+	15%

AFP—alpha-fetoprotein, β-hCG—beta-human chorionic gonadotropin, LDH—lactate dehydrogenase, GCTs—malignant germ cell tumors, ^a^ estimated.
